# Factors that influence the beta-diversity of spider communities in northwestern Argentinean Grasslands

**DOI:** 10.7717/peerj.1946

**Published:** 2016-04-21

**Authors:** Sandra M. Rodriguez-Artigas, Rodrigo Ballester, Jose A. Corronca

**Affiliations:** CONICET, IEBI-Facultad de Ciencias Naturales, Universidad Nacional de Salta, Salta, Argentina

**Keywords:** Geographic distance, Environmental heterogeneity, Climate, Corrientes-Argentina, Dispersion

## Abstract

Beta-diversity, defined as spatial replacement in species composition, is crucial to the understanding of how local communities assemble. These changes can be driven by environmental or geographic factors (such as geographic distance), or a combination of the two. Spiders have been shown to be good indicators of environmental quality. Accordingly, spiders are used in this work as model taxa to establish whether there is a decrease in community similarity that corresponds to geographic distance in the grasslands of the Campos & Malezales ecoregion (Corrientes). Furthermore, the influence of climactic factors and local vegetation heterogeneity (environmental factors) on assemblage composition was evaluated. Finally, this study evaluated whether the differential dispersal capacity of spider families is a factor that influences their community structure at a regional scale. Spiders were collected with a G-Vac from vegetation in six grassland sites in the Campos & Malezales ecoregion that were separated by a minimum of 13 km. With this data, the impact of alpha-diversity and different environmental variables on the beta-diversity of spider communities was analysed. Likewise, the importance of species replacement and nesting on beta-diversity and their contribution to the regional diversity of spider families with different dispersion capacities was evaluated. The regional and site-specific inventories obtained were complete. The similarity between spider communities declined as the geographic distance between sites increased. Environmental variables also influenced community composition; stochastic events and abiotic forces were the principal intervening factors in assembly structure. The differential dispersal capacity of spider groups also influenced community structure at a regional scale. The regional beta-diversity, as well as species replacement, was greater in high and intermediate vagility spiders; while nesting was greater in spiders with low dispersion capacity. Geographic distance, among other factors (climate, and active and passive dispersion capacity), explains assembly structure and the decrease spider community similarity between geographically distant sites. Spiders with the highest dispersal capacity showed greater species replacement. This may be due to the discontinuity (both natural and anthropic) of the grasslands in this ecoregion, which limits the dispersal capacity of these spiders, and their close dependence on microhabitats. The dispersal capacity of the least vagile spiders is limited by geographic distance and biotic factors, such as competition, which could explain the nesting observed between their communities.

## Introduction

The relative importance of local and regional environmental factors as controllers of local community assembly is a central question in ecology and biogeography (Bell, 2001; Hubbell, 2001; Ricklefs, 2004). Beta-diversity (*β*), defined as the spatial change in species composition (Whittaker, 1960), can be the result of the replacement of some species from one community to another; or the result of nesting that reflects a process of species loss (or gain) (Baselga, 2010; Baselga, 2012). Thus, beta-diversity can be considered a key concept to understand how is the local community assembly (Condit et al., 2002; Gering & Crist, 2002); the behaviour of ecosystems; their operation and for the conservation of biodiversity (Legendre, Borcard & Peres-Neto, 2005).

There is a growing interest in identifying which factors determine beta-diversity patterns, and how these influence assembly structure on different spatial scales (Carvalho et al., 2011). Several authors have associated beta-diversity with environmental or geographic factors, as well as combinations of the two (Tuomisto, Ruokolainen & Yli-Halla, 2003; Legendre, Borcard & Peres-Neto, 2005; Soininen, McDonald & Hillebrand, 2007; Qian, Badgley & Fox, 2009; Jiménez-Valverde et al., 2010; Carvalho et al., 2011), demonstrating that the importance of these factors depends on the group and taxonomic level studied, on spatial scale and the geographic region analysed.

Whittaker (1956) and Whittaker (1960) proposed the decline in similarity with increasing geographic distance. That is to say, the similarity in species composition between two sites diminishes as the geographic distance between them increases (Whittaker, 1956; Nekola & White, 1999). Soininen, McDonald & Hillebrand (2007) proposed three mechanisms which can act simultaneously and allow explain this phenomenon. The first suggests that similarity decreases with geographic distance because there is a corresponding increase in environmental dissimilarity that provokes greater species replacement. This mechanism supposes that species are distributed according to their distinct and specific requirements and to their tolerance for diverse environmental conditions (Nekola & White, 1999). A second mechanism suggests that the decline in similarity could be due to structural properties of the landscape that limit the range of dispersal. This proposal envisions a non-homogeneous landscape and the presence of barriers to dispersal (Garcillán & Ezcurra, 2003) that affect displacement. Finally, the decrease in similarity due to increasing distance between communities could be explained by a species own dispersal limit (neutral theory) within a homogeneous space (Hubbell, 2001).

Among arthropods, spiders have gained wide acceptance as indicators of environmental quality (Pinkus-Rendón, León-Cortés & Ibarra-Núñez, 2006); as such, their abundance, species richness and community structure are useful indicators of the biodiversity of the biocoenosis as a whole (Willett, 2001). Spider species richness has been correlated with latitude and mean annual temperature (Finch, Blick & Schuldt, 2008; Whitehouse et al., 2009), with habitat complexity and maximum regional temperature (Jiménez-Valverde & Lobo, 2007) as well as with altitudinal gradients (Chatzaki et al., 2005; Bowden & Buddle, 2010). Spiders respond quickly to brusque changes in habitat heterogeneity (Rubio, Corronca & Damborsky, 2008), and as a consequence of the narrow spatial scale in which spiders divide these biotic and abiotic changes, they become an appropriate species to evaluate patterns in diversity at a regional scale. As follows, a decrease in similarity between spider assemblages was recorded as climatic and geographic distance increased (Carvalho et al., 2011), although the response to the latter was weak in some cases (Jiménez-Valverde et al., 2010).

Spiders use different strategies to capture prey, this variety not only indicates their diversity, but can also reflect diversity at other trophic levels (Żmudzki & Laskowski, 2012). They are distributed across the majority of terrestrial ecosystems, as well as some freshwater ecosystems (Turnbull, 1973). Their wide distribution can be explained by the capacity to disperse passively through the air via silk threads (ballooning) (Brændegaard, 1937; Humphreys, 1987; Foelix, 2011) and occupy a variety of available niches. The distance that a spider can travel by this method depends primarily on body weight and wind, though under favourable conditions a spider could travel several hundred kilometres (Okuma & Kisimoto, 1981). The wide variety of body sizes and ecological strategies between spider families renders differences in their capacity to disperse passively (Bishop & Riechert, 1990; Suter, 1999). Consequently, supposing that ballooning is the primary method of long-distance dispersal (Bishop & Riechert, 1990; Thomas, Brain & Jepson, 2003), different families should have different beta-diversity patterns. Some vagile species can reach any available habitat under favourable environmental conditions, while other species with poor dispersal capabilities are unable to colonize all of the habitats in which they could survive (Araujo & Pearson, 2005; Steinitz et al., 2006).

In addition to the dispersal capacity, other factors can explain the diversity of spiders including their correspondence with the habitat heterogeneity (Uetz, 1991; Whitmore et al., 2002; Jiménez-Valverde & Lobo, 2007); understood the latter as the complexity of vertical and horizontal vegetation (Tews et al., 2004). Thus, heterogeneous habitats provide more available niches and many alternative ways of exploit environmental resources (Bazzaz, 1975). Habitat heterogeneity influences microclimate and determines the community of herbivorous species, which are spiders’ primary food source; and, consequently, it influences species composition and abundance in different habitats (Pearce et al., 2004). Moreover, climate factors such as temperature, humidity and precipitation (Bonte, Baert & Maelfait, 2002; Jiménez-Valverde & Lobo, 2007; Paredes Mungía, 2012), select the species that can live in each locality (Jiménez-Valverde et al., 2010).

Five ecoregions converge in Corrientes province, including the Campos & Malezales ecoregion (Burkart et al., 1999). This ecoregion is considered a prolongation of the Paranaense jungle and extends outward from the southeastern edge of Misiones province, encompassing an area of 30,000 km^2^. The landscape matrix, principally grassland, bestows the region with great potential for agriculture and cattle farming, the latter being the predominant anthropic activity in the region (Matteucci, 2012). Over the last ten years, multiple authors (Avalos et al., 2007; Rubio, Corronca & Damborsky, 2008; Avalos et al., 2009; Rubio & Moreno, 2010) have analysed spider fauna in the province however, only the recent study by Rodriguez Artigas (2014) has attempted to identify which factors influence spider communities at different spatial scales in the four ecoregions in Corrientes. Notwithstanding, that study did not consider geographic distance as a possible determinant in diversity patterns. For this reason, the present study seeks to examine the influence of geographical distance and the effect of environmental variables (climate and habitat heterogeneity) on spider diversity patterns of Campos & Malezales ecoregion. Likewise, this study evaluates whether the differences in the dispersal capacity of spider species influences their community structure at a regional scale. We expect that spider assemblages would differ between sites, and more so at more distant sites; demonstrating that the most vagile spider families are more dependent on environmental characteristics and that geographic distance would not be among the primary forces driving assembly structure. Moreover, it is expected that spiders with high vagility can reach to more available niches, showing a low beta diversity.

**Figure 1:**
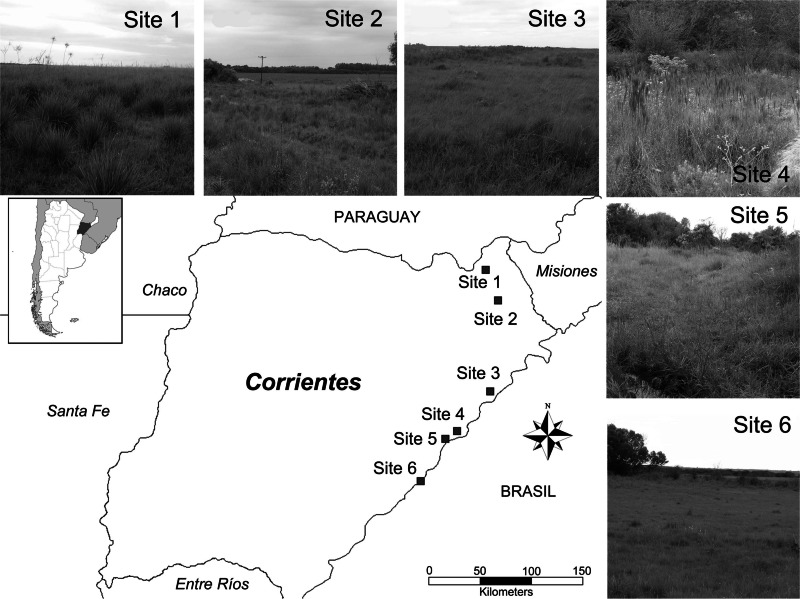
Studied area. Map of the area of study highlighting sampling sites.

## Materials and Methods

*Area of study:* Six sites that were geo-referenced of grasslands in eastern Corrientes province (Argentina) within the Campos & Malezales ecoregion were considered in this study. Sites were arranged from north to south between 27°33′–$29\textdegree 3{0}^{^{\prime}}\mathrm{S}$ and 56°14′–$58\textdegree 4{9}^{^{\prime}}\mathrm{W}$, separated by a distance no smaller than 13 km and reaching 250 km between the most distant pair (Fig. 1). This ecoregion is distinguished by its vegetation, the matrix consisting of vast grasslands of plain, periodically interrupted by relict patches of Paranaense jungle-like forest. The region has a humid subtropical climate with isohyets at 1,800 mm to the northeast and 1,300 mm to the southeast and uniform rain throughout the year. The mean temperature oscillates between 20 and 22 °C. There are periodic floods and fires, the latter used as a management tool that to regulate the dynamic vegetation of the ecoregion. The grasslands are low and dominated by *Andropogon, Aristida*, *Briza*, *Erianthus*, *Piptochaetium*, *Poa*, *Stipa*, *Paspalum*, *Axonpus* and *Panicum* species. The small patches of open forest that arise from the savannah are characterized by *Syagrus*, *Acacia*, *Allagoptera* and *Diplothemium* species (Matteucci, 2012).

*Sampling:* Samples were collected from vegetation with a G-Vac (garden vacuum) by the IEBI team (Institute for the Study of Invertebrate Biodiversity- FCN-U.N.Sa) between the spring of 2006 and autumn of 2007. In each collecting site, we selected two areas of 2 ha each, located one on the left and the other on the right side of the route, separating from the route border by no less than 150 m. In each area, 10 randomly samples were taken per season (Spring and Autumn). Each suction sample was spaced from another by not less than 50 m. Thus, a total of 40 suction samples was taken per collecting site. A single suction sample consisted of vacuuming an area of one square meter for one minute. The material collected was placed in polyethylene bags with 70% ethyl alcohol, labelled appropriately, and transported to the laboratory for analysis. Spiders were recorded in spreadsheets and all were classified by family following the keys available (Dippenaar-Schoeman & Jocqué, 1997; Ramírez, 1999; Ubick et al., 2005). Morphospecies were assigned according to the methodology proposed by Oliver & Beattie (1996) to those spiders that could not be identified to species taxonomic level. This information was incorporated into the database at IEBI (IEBIData) which includes digital pictures of somatic and external genital distinctive characters of each species/morphospecies that easily permit to identify individuals and assign them to a correct species morphospecies. Collected specimens were deposited in the IEBI-MCN Collection (Instituto para el Estudio de la Biodiversidad de Invertebrados-Museo de Ciencias Naturales, Universidad Nacional de Salta).

*Environmental variables:* To characterize each of the selected samples sites, information on eight bioclimatic variables were obtained from the WorldClim-Bioclim (www.worldclim.org) database, including temperature and precipitation; two variables associated with productivity (Normalized vegetation Index, NDVI) for the months in which samples were taken, which were obtained from Modis satellite images from NASA (http://modis.gsfc.nasa.gov/), and four variables, measured *in situ*, of local vegetation heterogeneity. The latter variables were quantified as a percentage vegetation per strata at 50 cm intervals, from ground level to 2 m, as described by Huang et al. (2011). This measurement was only taken in the autumn and, therefore, variables associated with environmental heterogeneity were only incorporated into analysis that included autumn.

*Selection of spider families with different dispersal capacities:* To test whether the differential dispersal capacities of spider families influence the structure of spider communities on a regional scale, six spider families were selected: Araneidae, Theridiidae, Thomisidae, Salticidae, Anyphaenidae and Miturgidae. The first two families build webs to capture prey; while the other four ones hunt on the ground and in the vegetation (Cardoso et al., 2011). Different spider families have distinct dispersal capacities, both active and passive. Araneidae and Theridiidae dispersal passively through the air (ballooning), which allows them to travel several hundred kilometres under favourable conditions (Okuma & Kisimoto, 1981). Thomisidae can also dispersal via ballooning (Foelix, 2011); however, like Salticidae (both foliage runners) they tend to dispersal on the ground (Liljesthröm et al., 2002). These families can rapidly run short distances and use this dispersal method widely (Dondale et al., 1970). Finally, Anyphaenidae and Miturgidae exclusively dispersal on the ground (Gertsch, 1979), and this dispersal is limited. Following this criteria and that outlined by Jiménez-Valverde et al. (2010), the families are grouped as: high vagility (Araneidae and Theridiidae); intermediate vagility (Thomisidae and Salticidae); and low vagility (Anyphaenidae and Miturgidae).

### Data analysis

*Inventory and alpha diversity*: Inventory completeness was evaluated using the non-parametric estimators of species richness Chao1 and ACE, which represent the lower and upper limits, respectively, of the species richness in communities with highly heterogeneous samples (Chao & Shen, 2012). These estimators were calculated with the program SPADE (Chao & Shen, 2009) and were used to calculate inventory completeness at different scales. The program PAST v2.17 (Hammer, Harper & Ryan, 2001) was used to run an analysis of similarities (ANOSIM) to test for statistically significant differences between spider assemblages at the sampling sites. To evaluate and compare the diversity between sites, the true observed diversity and the estimated diversity for each community were calculated using the Rényi one-parametric diversity index family (Tóthmérész, 1995) and the estimators Chao, Chao & Shen and MVUE to estimate zero, first, and second-order diversity, respectively. Both calculations were made using the PAST v2.17 (Hammer, Harper & Ryan, 2001) and SPADE (Chao & Shen, 2009) programs.

*Evaluation of geographic distance and environmental variables with respect to spider communities:* A simple Mantel test verified the relative importance of geographic distance on the patterns of beta-diversity obtained. The test considered a faunistic matrix (generated using Jaccard distance data as a measure of the similarity between spider communities between sites), and a geographic distance matrix (generated using site coordinates in WGS84 format and the geographic distance as a measure of distance between site pairs). The Mantel tests were performed with the PAST v2.17 (Hammer, Harper & Ryan, 2001) program, using Pearson’s correlation coefficient and 10,000 permutations to evaluate the statistical significance between matrices. The influence of geographical distance and environmental variables (climate and vegetation) on the distribution pattern of spiders was assessed by a variation partitioning procedure (Borcard, Legendre & Drapeau, 1992) using the package “Vegan” in the software R. Thus, the total variation of the abundance matrix was partitioned in its purely spatial, purely climate and purely vegetation complexity effects in the fraction explained by the correlation between them; and also by the residual fraction. Previously, principal coordinates of neighbour matrices (PCNM) was performed to obtain spatial variables. These variables, as environmental ones, were subjected to a run forward selection, using an analysis of canonical redundancy (RDA) (Legendre & Legendre, 1998) to select the variables that were included in the variation partitioning procedure (Blanchet, Legendre & Borcard, 2008). *β*_*RC*_ was calculated based on the Raup-Crick similarity, following the formula outlined by Chase et al. (2011). This measurement permitted the inference of events associated the dissimilarity between site pairs in accordance with the following interpretation: *β*_*RC*_ ≈ 0: Similarity${}_{\mathrm{observed}}\approx $ Similarity${}_{\mathrm{expected}}$ (stochastic events structure communities, high dispersion between sites); *β*_*RC*_ ≈ 1: Similarity${}_{\mathrm{observed}}$ < Similarity${}_{\mathrm{expected}}$ (differences in deterministic environmental filters between sites favours dissimilarity in species composition, biotic forces drive differentiation in adjacent communities); and *β*_*RC*_ ≈ − 1: Similarity${}_{\mathrm{observed}}$ > Similarity${}_{\mathrm{expected}}$ (shared deterministic environmental filters generate high similarity between sites as a result of abiotic factors).

The same methodology above described was applied to the question of correlation between the faunistic, geographic distance and environmental matrices for each of the family groups selected, taking into account dispersal capacity and the importance of ballooning (wind dispersal) as a dispersal technique in the structuring of spider communities at a regional scale.

*Dispersal and beta-diversity:* The contribution of beta-diversity in the regional diversity (gamma) of the groups with different dispersal capacities was calculated from a multiplicative partition of diversity using the program PARTITION 3.0 (Veech & Crist, 2009), where: *γ* = *α*_1_ (within the samples) × *β*_1_ (between samples) × *β*_2_ (between regional sites). The latter observed *β* value was collated with the value expected with random distribution (Crist et al., 2003), and compared between groups. Finally, the total beta-diversity of each group of families with distinct dispersal capacities was partitioned into its components, nesting and species replacement, as described in the Sørensen index. The R “betapart” pack provided by Baselga et al. (2013) was used for this analysis.

## Results

### Inventory and alpha diversity

A total of 3.873 adult and immature spiders, belonging to 251 species/morphospecies and 27 families, were collected. Araneidae, Theridiidae and Oxyopidae were the dominant families, representing 29.43%, 19.42% and 19.10%, respectively, of total abundance; while Araneidae and Salticidae had greater species richness (*S* = 52 and *S* = 40, respectively). The performance of the non-parametric estimators of species richness found that the inventory reached 74% of the value estimated by Chao1 (Table 1).

**Table 1:** Diversity parameters. The abundance and species richness of spider species, non-parametric estimator values (Chao1 y ACE), of the inventory completeness and the true observed and estimated diversity.

		Site 1	Site 2	Site 3	Site 4	Site 5	Site 6	TOTAL
Richness		40	80	110	82	84	103	251
Abundance		102	803	703	480	843	942	3,873
Chao1		67.56	121.88	175.03	122.83	114.15	166.18	339.11
ACE		72.6	138.7	182.0	129.3	121.7	224.9	342.6
Inventory completeness (Chao1)		59.20%	65.64%	62.84%	66.76%	73.58%	61.98%	74.02%
Observed diversity	${}^{0}{D}_{(\mathrm{Sobs})}$	40	80	110	82	84	103	–
	${}^{1}{D}_{(\mathrm{Shannonentropy})}$	27.13	13.20	28.22	29.50	31.59	20.59	–
	${}^{2}{D}_{(\mathrm{Gini- SimpsonIndex})}$	19.12	4.61	11.12	12.78	19.95	8.62	–
Estimated diversity	${}^{0}{D}_{(\mathrm{Chao})}$	67.6	121.9	175.0	122.8	114.2	166.2	–
	${}^{1}{D}_{(\mathrm{Chao{\XMLAMP} Shen})}$	37.70	15.31	34.49	35.12	34.73	24.17	–
	${}^{2}{D}_{(\mathrm{MVUE})}$	23.31	4.63	11.28	13.10	20.41	8.69	–

Spider communities differed between pair of sites (Anosim: *R* > 0.63, *p* < 0.01). All sites had a specific richness equal to or greater than 80 species, except for site 1 (Table 1). The inventory of each site was unable to capture the total spider diversity present in each site, though completeness values were good and were near to or greater than 60% of the Chao1 estimate (Table 1). The true observed and estimated diversity parameters indicate different community behaviour in rare and dominant species; consequently, under these circumstances sites were not able to compare in this respect (Table 1).

### Beta-diversity: similarity decreases with geographic distance

**Figure 2:**
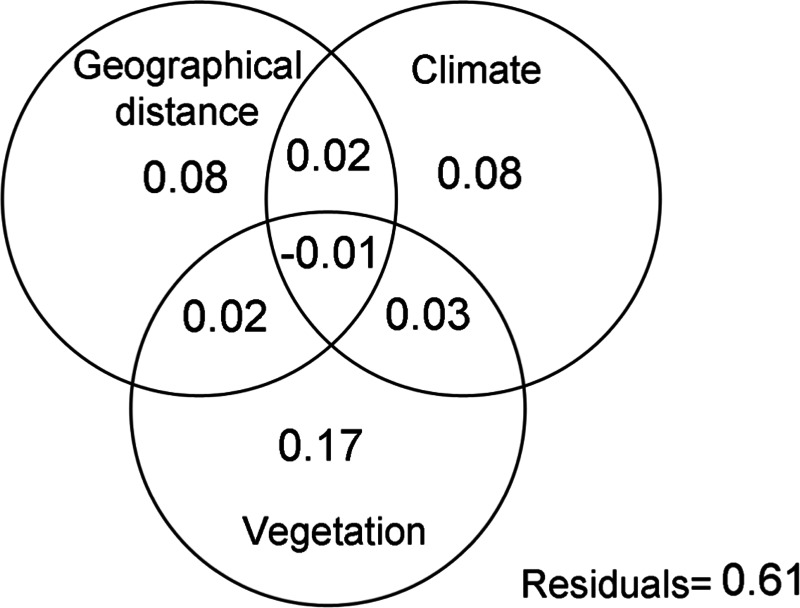
Variation partitioning. Variation partitioning showing the relative influence of the geographical distance, climate and vegetation complexity variables on the spider assemblages in grassland of Campos & Malezales ecoregion (Corrientes-Argentina).

The Mantel test (*R* = 0.53; *p* < 0.05), demonstrated that geographic distance had a direct effect of the similarity of spider assemblages, confirming that these communities become less similar as the distance between them increases. This variable, together with environmental ones explained 39% of the changes in the composition of spider assemblages (Fig. 2). The variation mainly corresponded to the pure effects of vegetation complexity (17%), the latter with climate explained 28% of the composition changes. The analysis of Raup-Crick similarity based on standardized values also supported these findings, showing that deterministic abiotic forces followed by stochastic events are important factors that contribute to the structure of the spider communities considered in this study (Table 2).

**Table 2:** Raup–Crick values. Raup–Crick values are indicated below the diagonal and modified Chase et al. (2011) values above the diagonal (light grey =*βRC* ≈ 0, black =*βRC* ≈ 1, dark grey =*βRC* ≈ − 1).

	Site 1	Site 2	Site 3	Site 4	Site 5	Site 6
Site 1	**–**	0.773	−0.664	−0.915	−0.247	−0.424
Site 2	0.887	**–**	0.531	0.331	−0.971	−0.721
Site 3	0.168	0.766	**–**	0.846	−0.939	−0.956
Site 4	0.043	0.666	0.077	**–**	0.005	−0.353
Site 5	0.377	0.015	0.031	0.503	**–**	−0.674
Site 6	0.288	0.140	0.022	0.324	0.163	**–**

**Notes.**

*βRC* ≈ 0: Similarity_*observed*_ ≈ Similarity_*expected*_ (stochastic events structure communities, high dispersion between site); *βRC* ≈ 1: Similarity_*observed*_ < Similarity_*expected*_ (differences in deterministic environmental filters between sites favors dissimilarity in species composition, biotic forces drive differentiation in adjacent communities); *βRC* ≈ − 1: Similarity_*observed*_ > Similarity_*expected*_ (shared deterministic environmental filters generate high similarity between sites as a result of abiotic factors).

### Beta-diversity: the relationship between dispersion and beta-diversity

**Figure 3:**
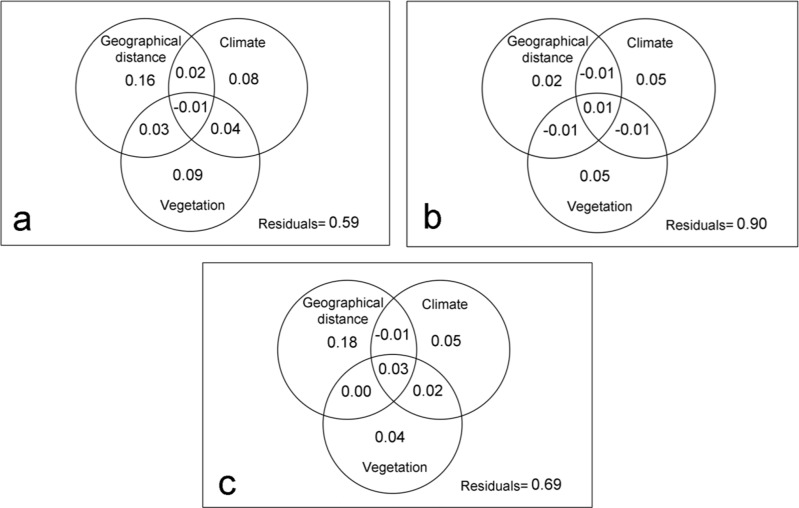
Variation partitioning by families with different dispersal capacity. Variation partitioning showing the relative influence of the geographical distance, climate and vegetation complexity variables in spider families with high (A), intermediate (B) and low (C) dispersal capacity.

With respect to the dispersal capacity of spider families, the geographic distance explained 16% of the variation in the composition of the spider assemblages with high dispersal capacity (Fig. 3A), however, it was not significant according to the Mantel test (*R* = 0.44; *p* > 0.05). Meanwhile, 21% of the changes in the composition of communities can be attributed to the purely climate and purely vegetation complexity effects. This percentage rises to 25% if it also considers its spatial structure. Thus, even though stochastic event are important, the deterministic abiotic forces were the key factor driving spider community structure (Table 3).

**Table 3:** Raup–Crick values by families with different dispersal capacity. Raup–Crick values are indicated below the diagonal and modified Chase et al. (2011) values above the diagonal (light grey =*βRC* ≈ 0, black =*βRC* ≈ 1, dark grey =*βRC* ≈ − 1) in families with high intermediate and low vagility.

	Site 1	Site 2	Site 3	Site 4	Site 5	Site 6
**High**						
Site 1	**–**	0.539	−0.326	−0.923	−0.120	−0.526
Site 2	0.770	**–**	0.512	−0.744	−0.609	0.165
Site 3	0.337	0.756	**–**	−0.903	−0.629	0.522
Site 4	0.039	0.128	0.049	**–**	0.359	−0.623
Site 5	0.440	0.196	0.186	0.680	**–**	−0.085
Site 6	0.237	0.583	0.761	0.189	0.458	**–**
**Medium**						
Site 1	**–**	−0.465	−0.519	−0.537	−0.879	−0.739
Site 2	0.268	**–**	0.163	0.856	−0.835	−0.379
Site 3	0.241	0.582	**–**	−0.225	0.581	−0.786
Site 4	0.232	0.929	0.388	**–**	0.685	−0.848
Site 5	0.061	0.083	0.210	0.843	**–**	0.580
Site 6	0.131	0.311	0.107	0.076	0.210	**–**
**Low**						
Site 1	**–**	0.895	0.560	0.753	0.643	0.542
Site 2	0.948	**–**	0.065	0.954	−0.276	0.008
Site 3	0.780	0.533	**–**	0.095	0.575	−0.877
Site 4	0.877	0.977	0.548	**–**	−0.577	−0.467
Site 5	0.822	0.362	0.788	0.212	**–**	0.012
Site 6	0.771	0.504	0.062	0.267	0.506	**–**

**Notes.**

*βRC* ≈ 0: Similarity_*observed*_ ≈ Similarity_*expected*_ (stochastic events structure communities, high dispersion between site); *βRC* ≈ 1: Similarity_*observed*_ < Similarity_*expected*_ (differences in deterministic environmental filters between sites favors dissimilarity in species composition, biotic forces drive differentiation in adjacent communities); *βRC* ≈ − 1: Similarity_*observed*_ > Similarity_*expected*_ (shared deterministic environmental filters generate high similarity between sites as a result of abiotic factors).

In the intermediate vagile spider families, the geographical distance had little influence in the dissimilarity of spider assemblages (Mantel test: *R* = 0.31; *p* > 0.05) (Fig. 3B), being environmental variables the most important in structuring community. However, the model only explained 10% of the changes in spider assemblages indicating that other factors, mainly biotic according to *β*_*RC*_ values, could influence the distribution pattern of the species (Table 3).

Geographic distance did an important role in structuring the assemblages of families with a low dispersal capacity (Mantel test: *R* = 0.49; *p* < 0.05), explaining 18% of the variation of the assemblages (Fig. 3C). Environmental variables also influenced on the community dissimilarity, although the biotic forces and stochastic factors would be the most important for structuring the communities of the least vagile spider (Table 3).

**Figure 4:**
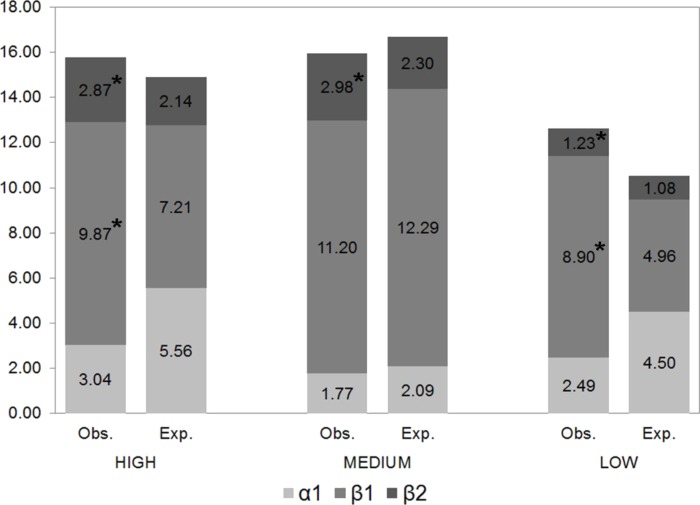
Partition of gamma diversity of spider diversity. Multiplicative partition of the diversity of families with high, intermediate and low dispersal capacity (* indicates statistically significant differences between observed and expected values).

Regional beta-diversity (*β*_2_) was greater in families with a high dispersal capacity and lesser in less vagile families, exceeding the values expected by chance by 34%, 29% and 14%, respectively (Fig. 4). *β*_2_ (between sites) diversity significantly exceeded expected diversity values, indicating that these species were not randomly distributed. Likewise, the differences between the expected and observed *β*_1_ (between samples) diversity values were statistically significant for the families with the greatest and lowest dispersal capacity, revealing their capacity to occupy different available niches.

Dissimilarity (*β*_*SOR*_) and species replacement between sites (*β*_*SIM*_) were greater for spiders with high and intermediate dispersal capacity (Table 4), representing 91% and 87%, respectively, of the total beta-diversity. The least vagile families had the least species replacement but the highest nesting value (25% of the observed dissimilarity) (Table 4).

## Discussion

Spider species richness in the grassland of the Campos & Malezales ecoregion is high; 251 species/morphospecies from 27 Araneomorphae families were recorded, which corresponds to 90% of the total number of families recorded in Corrientes province (Rodriguez Artigas, 2014). Our results are comparable to those obtained by other authors in the same province who have used intensive sampling methods. Rubio, Corronca & Damborsky (2008) reported 28 spider families in forests and grasslands in the Mburucuyá National Park; while Avalos et al. (2009) found 33 families in samples obtained from the Iberá Provincial Reserve. In this work, four of the families corresponded to orbicular spiders, three of which were also described in the forests and grasslands of the same ecoregion by Rubio & Moreno (2010).

**Table 4:** Partition of the total beta diversity into its components. Partition of the total beta-diversity into its components, species replacement (${\beta }_{\mathrm{SIM}}$) and nesting (${\beta }_{\mathrm{SNE}}$) for families with varying vagility (different letters indicate statistically significant differences between groups *p* < 0.05).

VAGILITY	*β*_*SIM*_	*β*_*SNE*_	*β*_*SOR*_
High	0.676${}^{\mathrm{a}}$	0.069${}^{\mathrm{a}}$	0.745${}^{\mathrm{a}}$
Medium	0.665${}^{\mathrm{a}}$	0.100${}^{\mathrm{b}}$	0.765${}^{\mathrm{a}}$
Low	0.531${}^{\mathrm{b}}$	0.177${}^{\mathrm{c}}$	0.709${}^{\mathrm{b}}$

Although it is difficult to sample the totality of a spider assemblage due to the large number of rare species collected (Haddad et al., 2010), a large proportion (74%) of the species present in the Campos & Malezales grasslands were captured in this study; this percentage was near to or greater than 60% for each site included in this study. These results indicate good (Cardoso, 2009) and complete inventories, since the percentage exceeds 50% of the estimated total number of species (Chao et al., 2009).

Whittaker (1956) and Whittaker (1960) proposed that geographic distance influences the structure of the spider assemblages studied, showing a decrease in the similarity of spider communities as geographic separation increases. Similar results were reported by Carvalho et al. (2011) in the Portuguese region of the Iberian Peninsula. The effect of geographical distance could be explained by the first mechanism proposed by Soininen, McDonald & Hillebrand (2007), because environmental variables, also explained the species turnover, being the habitat heterogeneity (complexity of vegetation) the most relevant. Thus, stochastic and deterministic events like dispersal and abiotic factors, respectively, would be the principle intervening forces on community structure, as was verified by the variation partitioning and *β*_*RC*_ values.

The results obtained here counter previous findings from a spider study in Madrid Province (Spain), in which Jiménez-Valverde et al. (2010) observed that geographic distance was not an important factor explaining the differences in community composition. However, other empiric studies revealed that the relationship between similarity and geographic distance is strongly dependent on the extent of the area of study (Martiny et al., 2011; Soininen et al., 2011; Steinbauer et al., 2012). This could explain the discrepancy between the results obtained here and in the Spanish study, since the distance between the most distant sites in our study was double that of the Jiménez-Valverde et al. (2010) study.

Although the environmental variables considered have an effect on the similarity of spider assemblages.Thus, as suggested by Jiménez-Valverde et al. (2010) climate conditions select which spider species can live in a given locality, but not how many, and that habitat complexity is the principal factor determining the number of species that a locality can support.

Furthermore, the differential dispersal capacities of the spiders can influence community structure at a regional scale. Araneidae and Theridiidae contributed the most to regional beta-diversity and, together with Salticidae and Thomisidae, were the most dissimilar and with a higher species replacement between communities. These results are not consistent with the findings of Jiménez-Valverde et al. (2010), who considered a different set families in their analysis (Araneidae = high vagility; Thomisidae and Salticidae = intermediate vagility and Gnaphosidae = low vagility). The dispersal capacity a species can be affected by habitat fragmentation (Garcillán & Ezcurra, 2003), increasing beta-diversity between communities. This could explain our results, since the matrix of the grasslands in the Campos & Malezales ecoregion contains remnant patches of Paranaense jungle (Matteucci, 2012) interspersed in a landscape where humans undertake a variety of activities (ranching, agriculture and road-building, among others). This could interrupt aerial dispersal increasing beta-diversity of the most vagile spider communities.

The geographic distance was not a relevant factor structuring the assemblages intermediate vagility spiders. In this group, the dissimilarity between communities might be determined by other abiotic factors not included in this analysis. For their part, the vegetation complexity and climatic distance explained the composition of the high-vagility spider assemblages; suggesting, as mentioned by Jiménez-Valverde & Lobo (2007), that spider diversity is principally determined by vegetation complexity and the climatic conditions of a given site. As in the general model, geographic distance generates an environmental dissimilarity (Soininen, McDonald & Hillebrand, 2007) that explains the changes in species composition of the families of more vagile spiders. The spiders of these families dispersal aggregately on a local scale, showing a close dependence on micro-habitats, which are necessary to fasten webs (Simó et al., 2011) and serve as sites of refuge (Jiménez-Valverde & Lobo, 2007). These spiders select micro-habitats that favour prey availability (Sunderland & Samu, 2000) or minimize competitive exclusion and cannibalism (Viera, 2011).

Stochastic factors like dispersal are important in structuring the assemblages of low vagility spider families. However, this last factor (dispersal) would be limited by the separation between sites, as was demonstrated in these results. Thus, the dispersal of this group of spiders is affected by the distance between sites responding to second and third mechanism proposed by Soininen, McDonald & Hillebrand (2007). For their part, biotic factors such as competition could lead to species exclusion (Wise, 2006) and would be relevant to this group of spiders; this would explain the greater degree of nesting between these communities with respect to families with high and intermediate dispersal capacity.

## Conclusions

Our results reflect that the grasslands in the Campos & Malezales ecoregion support a great diversity of spider species that vary between sites. This indicates that despite a homogeneous appearance, grasslands are a heterogeneous habitat from a spider perspective (Ferro & Romanowski, 2012). Geographic distance does influence the decrease in spider community similarity, but it is not the only force explain beta-diversity between distant sites. There are other factors that contribute to the structuring of the local and regional spider assemblages: such as climatic factors, vegetation complexity, and dispersal acting differently according to the studied group. Dispersal has a differential effect on distinct spider assemblages; this effect is heavily influenced by the active and passive dispersal capacity of each assemblages constituents. Future studies that evaluate additional variables may be necessary to identify additional forces that determine the structure of the spider communities in these grasslands, particularly of intermediate vagility spiders, which are important for maintaining diversity in their communities.

## Supplemental Information

10.7717/peerj.1946/supp-1 Supplemental Information 1 Spider abundance of the species/morphospecies by sites Table of spider species/morphospecies by sites collected in Corrientes grassland, Argentina (2006–2007).

## Additional Information and Declarations

### Data Availability

The following information was supplied regarding data availability: The raw data has been supplied as a Supplemental Dataset.
