# Supplementary material for: Factors that influence the beta-diversity of spider communities in northwestern Argentinean Grasslands
Source: PeerJ. 2016 Apr 21;4:e1946. doi: 10.7717/peerj.1946 (PMC4846805; doi:10.7717/peerj.1946)
Supplement: Supplemental Information 1 — Table of spider species/morphospecies by sites collected in Corrientes grassland, Argentina (2006–2007). [file peerj-04-1946-s001.pdf]

Families	Morpho	Species	Site 1	Site 2	Site 3	Site 4	Site 5	Site 6
Anyphaenidae	Ar116		0	0	0	1	0	0
	Ar145		12	0	0	0	0	1
	Ar197		1	1	0	1	0	0
	Ar205		0	0	1	0	0	0
	Ar298		0	0	0	0	1	0
	Ar305		0	0	0	0	2	0
	Ar362		0	0	0	0	0	1
	Ar363		0	0	0	0	0	2
	Ar373		0	0	0	0	1	0
	Ar402		0	0	0	2	7	4
	Ar475		0	0	1	0	0	0
	Ar482		0	0	2	0	0	2
	Ar520	*Arachosia* sp.	0	0	0	2	0	0
	Ar545		0	0	1	0	0	0
	Ar559		0	0	2	0	0	0
	Ar561		0	0	0	0	0	2
	Ar618		0	3	7	2	0	0
	Ar660		0	0	0	0	1	0
	Ar852		0	0	2	0	0	0
Araneidae	Ar060	*Parawiixia bistriata* (Rengger, 1836)	1	0	0	0	0	0
	Ar103	*Scoloderus sp.*	3	0	0	2	0	2
	Ar139	*Argiope argentata* (Fabricius, 1775)	0	0	0	0	2	0
	Ar184	*Ocrepeira hirsuta* (Mello- Leitao, 1942)	0	0	0	4	50	8
	Ar187	*Araneus* sp.	0	1	1	2	0	0
	Ar195	*Mastophora reimoseri* Levi, 2003	0	0	0	3	0	0
	Ar198	*Alpaida trispinosa* (Keyserling, 1879)	2	0	1	1	1	0
	Ar207	*Alpaida leucogramma* (White, 1841)	0	0	0	0	0	1
	Ar212		0	0	2	0	0	0
	Ar222	*Metepeira* sp.	0	14	12	12	39	28
	Ar233	*Alpaida* sp.	0	0	0	0	4	0
	Ar249	*Araneus* sp.	0	4	5	0	0	4
	Ar251	*Ocrepeira venustula* (Keyserling, 1879)	4	1	0	0	0	0
	Ar254	*Larinia directa* (Hentz, 1847)	0	1	0	0	0	0
	Ar255	*Araneus* sp.	0	0	0	0	0	0
	Ar268	*Metapeira galathea* (Thorell, 1891)	1	0	3	1	0	6
	Ar284	*Alpaida* sp.	1	1	6	3	0	0
	Ar288	*Larinia t-notata* (Tullgren, 1905)	2	355	70	71	88	142
	Ar299	*Araneus sp.*	0	0	1	0	0	0
	Ar300	*Argiope trifasciata* (Forsskal,1775)	8	15	50	10	6	40
	Ar328		0	49	1	0	3	0
	Ar331	*Alpaida bicornuta* (Taczanowski, 1878)	0	0	0	1	2	0
	Ar392	*Gea heptagon* (Hentz, 1850)	0	12	9	11	1	22
	Ar398	*Araneus guttatus* (Keyserling, 1865)	6	2	0	6	17	12
	Ar413		0	0	0	0	0	1
	Ar414	*Araneus unanimus* (keyserling, 1879)	0	1	0	0	0	0
	Ar417		0	1	0	1	0	1
	Ar423		1	0	3	6	0	2
	Ar429	*Acasecia villalobosi* Glueck, 1994	0	0	0	0	0	2
	Ar444		0	0	2	0	0	2
	Ar449		0	0	1	1	0	1
	Ar455		0	0	0	0	0	0
	Ar456		2	0	2	0	0	0
	Ar460		0	1	0	0	0	0
	Ar465		0	3	0	0	0	0
	Ar489	*Metazigia voluptifica* (Keyserling, 1892)	0	1	1	0	0	0
	Ar501	*Alpaida* sp.	0	17	23	0	0	2
	Ar516	*Pronus tuberculifer* Keyserlin, 1881	0	0	1	1	0	0
	Ar573		0	4	0	0	0	1
	Ar594		0	0	0	0	0	1
	Ar611		0	16	1	0	0	2
	Ar613		0	13	69	0	3	11
	Ar614		0	4	3	1	0	0

	Ar626		0	2	0	0	0	0
	Ar631		5	0	0	3	3	0
	Ar634		0	1	1	0	0	2
	Ar745		1	0	0	1	0	0
	Ar821		0	1	3	0	1	1
	Ar847		0	3	1	0	0	0
	Ar855		0	3	0	11	0	0
	Ar879		1	2	0	0	0	0
	Ar901		0	0	1	0	0	0
**Clubionidae**	Ar218		0	3	3	0	0	0
	Ar304		0	3	3	0	0	1
	Ar826		0	0	0	0	4	0
**Corinnidae**	Ar129		0	0	3	0	1	1
	Ar625		0	1	0	0	0	0
	Ar644		0	0	0	0	0	0
	Ar875		0	0	8	0	1	1
**Deinopidae**	Ar768		0	1	4	0	0	0
**Dictynidae**	Ar237		1	2	32	2	4	9
	Ar367		0	2	1	0	0	0
**Gnaphosidae**	Ar290	*Neodrassex cachimbo* Ott, 2013	0	0	1	0	0	2
	Ar548		0	0	0	0	2	0
	Ar617		0	1	0	0	0	0
	Ar652		0	0	0	2	0	0
	Ar844		0	0	1	0	2	0
	Ar846		0	0	3	0	0	0
	Ar851		0	85	170	22	47	104
**Hahniidae**	Ar056		0	0	2	0	0	0
	Ar756		0	0	0	0	0	1
**Linyphiidae**	Ar010		0	0	0	1	0	0
	Ar017	*Meioneta* sp.	0	0	0	0	0	0
	Ar030		0	2	2	1	2	8
	Ar097		2	0	1	1	0	0
	Ar202		0	7	5	18	5	23
	Ar246		0	0	0	0	4	1
	Ar253		0	0	2	0	0	0
	Ar267		1	0	3	0	20	2
	Ar283		0	0	0	0	0	1
	Ar293		0	0	0	0	0	1
	Ar599		0	0	0	0	0	0
	Ar610		0	0	0	0	2	0
	Ar619		1	5	4	5	7	0
	Ar706		0	1	0	1	0	0
	Ar876		0	0	1	0	0	0
**Lycosidae**	Ar007		0	46	11	8	24	30
	Ar459		0	2	0	5	4	0
	Ar483		0	0	0	0	1	0
	Ar515		0	0	0	0	1	0
	Ar562		0	3	0	1	0	1
	Ar622		2	1	0	0	0	0
	Ar800		0	4	0	0	0	0
**Micropholcommatidae**	Ar247		0	0	0	0	2	1
**Mimetidae**	Ar033		0	2	19	1	10	14
	Ar877		0	0	0	1	4	1
	Ar878		1	0	0	0	3	0
**Miturgidae**	Ar119		0	0	0	0	2	3
	Ar191		0	0	0	0	0	1
	Ar239		0	0	0	0	1	0
	Ar324		0	0	0	0	0	1
	Ar667		0	0	0	1	0	0
**Mysmenidae**	Ar329		0	0	1	0	62	4
	Ar860		0	2	2	1	2	0
**Oonopidae**	Ar605		0	1	0	0	0	1
	Ar194		7	0	0	12	68	0

**Oxyopidae**	Ar368		0	0	1	0	0	0
	Ar854		0	4	0	2	0	1
**Philodromidae**	Ar075		0	0	0	0	1	0
	Ar085		0	2	1	4	3	0
	Ar241	*Paracleocnemis* sp.	3	0	0	0	0	0
	Ar261		0	0	0	1	0	0
	Ar323		0	0	0	0	1	0
	Ar361		0	0	2	0	0	1
	Ar522		0	0	0	0	1	1
	Ar722		0	0	0	2	1	0
**Pholcidae**	Ar245	*Aymaria calilegua* Huber, 2000	0	1	0	0	0	0
	Ar682	*Mesabolivar uruguayensis* Machado, Laborda, Simó & Brescovit, 2013	0	4	0	1	0	0
**Pisauridae**	Ar213		0	1	2	0	0	0
	Ar262		0	0	0	0	0	0
	Ar303		0	0	1	0	0	1
	Ar380		0	0	0	0	0	1
	Ar426		1	0	0	0	0	0
	Ar586		0	0	0	2	0	2
	Ar700		0	0	0	2	0	6
	Ar858		0	0	0	0	0	2
**Prodidomidae**	Ar857		0	0	0	0	6	0
**Salticidae**	Ar040		0	0	0	1	0	0
	Ar094		0	0	1	0	2	2
	Ar123		0	0	0	0	0	1
	Ar163		0	0	0	0	0	1
	Ar189	*Chira micans* (Simon, 1902)	1	1	0	0	0	3
	Ar252		1	2	0	0	0	0
	Ar260		0	0	0	0	4	0
	Ar263		0	0	1	0	0	0
	Ar264		0	0	0	0	1	0
	Ar265	*Semora* sp.	0	0	2	0	0	0
	Ar266		1	0	1	0	0	0
	Ar287		1	0	4	2	1	0
	Ar294		0	1	0	1	0	3
	Ar295		0	0	0	1	0	1
	Ar309		2	1	1	0	0	0
	Ar330		0	0	1	1	0	0
	Ar345		0	1	14	4	0	0
	Ar348		0	0	0	11	0	0
	Ar381		0	0	0	0	0	5
	Ar384		0	0	0	0	4	0
	Ar395		0	0	1	0	0	0
	Ar408		0	0	0	1	0	3
	Ar411		0	0	0	0	1	0
	Ar433		0	0	1	1	0	0
	Ar447		2	1	0	0	0	0
	Ar547		0	1	3	0	0	0
	Ar553		0	1	2	0	0	4
	Ar554		0	1	0	0	0	0
	Ar582		0	0	14	0	0	1
	Ar584	*Euophrys* sp.	1	0	1	6	0	4
	Ar612		0	0	0	8	6	6
	Ar616		0	1	0	0	0	0
	Ar649		0	0	0	1	2	7
	Ar650		0	3	1	0	1	0
	Ar658	*Metaphidippus cupreus* F. O. Pickard-Cambridge, 1901	0	0	0	0	7	0
	Ar666		0	0	1	0	0	0
	Ar694		0	0	0	1	0	0
	Ar823		3	1	0	0	0	3
	Ar831		0	7	2	13	43	254
	Ar843		0	0	0	0	1	1
**Senoculidae**	Ar567		0	0	0	0	11	1
	Ar824		0	0	0	1	0	3

**Tetragnathidae**	Ar339		2	0	0	0	0	1
	Ar354		11	13	3	5	14	28
	Ar383		3	0	0	0	0	1
	Ar607		0	2	3	0	3	1
	Ar759		0	0	0	1	0	0
	Ar902		0	0	0	0	0	1
**Theridiidae**	Ar037		0	3	7	0	0	4
	Ar047		0	10	1	0	0	2
	Ar051		0	15	7	18	21	11
	Ar088		0	0	1	5	0	2
	Ar109		0	3	0	0	0	0
	Ar200		0	1	0	2	0	1
	Ar201		0	0	2	0	0	0
	Ar226		1	1	0	0	0	0
	Ar259		0	0	1	2	0	0
	Ar325		0	0	2	0	0	1
	Ar340		0	0	2	0	4	0
	Ar360		0	0	1	0	2	0
	Ar387		0	0	0	0	1	0
	Ar393		0	0	0	0	8	2
	Ar401		0	0	1	6	48	6
	Ar407		0	0	1	1	7	0
	Ar412		1	0	1	0	0	0
	Ar458		0	0	0	0	0	2
	Ar463		0	0	1	0	1	0
	Ar508		0	0	0	0	25	0
	Ar546		0	1	9	2	1	0
	Ar555		0	3	0	0	0	0
	Ar593		0	2	1	0	0	0
	Ar603		0	0	1	0	0	0
	Ar604		0	0	0	3	0	0
	Ar620		0	0	0	0	1	0
	Ar641		0	0	0	0	1	0
	Ar654		0	0	0	1	0	0
	Ar673		0	0	1	0	0	1
	Ar712		0	0	5	0	0	0
	Ar758		0	0	0	0	0	1
	Ar856		0	0	0	3	0	1
	Ar870		1	0	0	0	0	1
	Ar904		0	0	1	0	0	0
**Theridiosomathidae**	Ar154		0	1	6	0	0	1
	Ar583		0	0	1	0	0	0
**Thomisidae**	Ar029		0	0	1	0	0	0
	Ar039		0	0	1	0	0	0
	Ar065		0	0	2	12	4	1
	Ar082	*Misumenops* sp.	0	0	1	0	0	0
	Ar192	*Misumenops* sp.	0	0	3	0	11	1
	Ar221		0	0	2	0	0	0
	Ar240		0	0	0	1	0	0
	Ar285		0	0	0	1	0	0
	Ar289		0	0	3	0	0	0
	Ar313		0	0	1	6	0	0
	Ar350		0	0	1	0	0	0
	Ar377		0	8	0	101	74	33
	Ar424		1	0	0	0	1	0
	Ar560		0	0	0	0	1	0
	Ar651		0	0	0	0	1	0
	Ar659		0	0	0	0	0	0
	Ar727		0	0	0	0	0	0
	Ar836		0	0	0	0	0	1
	Ar841		0	0	0	0	0	1
**Uloboridae**	Ar385		0	0	0	0	0	1
